# GABAergic Neuronal Precursor Grafting: Implications in Brain Regeneration and Plasticity

**DOI:** 10.1155/2011/384216

**Published:** 2011-06-20

**Authors:** Manuel Alvarez Dolado, Vania Broccoli

**Affiliations:** ^1^Department of Cell Therapy and Regenerative Medicine, Andalusian Center for Molecular Biology and Regenerative Medicine (CABIMER), 41092 Seville, Spain; ^2^Stem Cell and Neurogenesis Unit, Division of Neuroscience, San Raffaele Scientific Institute, 20132 Milan, Italy

## Abstract

Numerous neurological disorders are caused by a dysfunction of the GABAergic system that impairs or either stimulates its inhibitory action over its neuronal targets. Pharmacological drugs have generally been proved very effective in restoring its normal function, but their lack of any sort of spatial or cell type specificity has created some limitations in their use. In the last decades, cell-based therapies using GABAergic neuronal grafts have emerged as a promising treatment, since they may restore the lost equilibrium by cellular replacement of the missing/altered inhibitory neurons or modulating the hyperactive excitatory system. In particular, the discovery that embryonic ganglionic eminence-derived GABAergic precursors are able to disperse and integrate in large areas of the host tissue after grafting has provided a strong rationale for exploiting their use for the treatment of diseased brains. GABAergic neuronal transplantation not only is efficacious to restore normal GABAergic activities but can also trigger or sustain high neuronal plasticity by promoting the general reorganization of local neuronal circuits adding new synaptic connections. These results cast new light on dynamics and plasticity of adult neuronal assemblies and their associated functions disclosing new therapeutic opportunities for the near future.

## 1. Introduction

*γ*-Aminobutyric acid (GABA) is the major inhibitory neurotransmitter in the central nervous system (CNS), playing a key role in the balance between inhibitory and excitatory circuits [1, 2]. Therefore, it is not surprising that dysfunctions in the GABAergic system lead to pathological conditions including hypokinetic diseases such as Parkinson's disease (PD), and hyperkinetic diseases, such as Huntington's disease (HD), when disruption of the GABAergic system occurs in the basal ganglia [3–5]. Epilepsy, a pathology characterized by uncontrolled hyperactivity, is also tightly linked to deficits in GABA levels, as well as alterations in its synthesis, secretion, and reuptake, or reductions in the number of GABAergic interneurons [6–8]. 

Almost 25 years ago, it was already postulated that controlling GABA delivery to specific brain areas should benefit each of these diseases [9, 10]. Cell transplantation is a powerful tool to introduce a new source of GABA and may allow reconstitution of neural circuits in the diseased brain [11, 12]. To be successful, grafted cells should possess the ability to disperse through affected areas and differentiate into fully mature neurons expressing appropriate neurotransmitters, in this case GABA. Ideally, these cells should also functionally integrate and modulate circuitry activity in the damaged host brain; for instance, affecting its plasticity. Since the pioneer works from Lindvall and Björklund [9] and Isacson et al. [10], several transplantation assays with different GABA-producing cell types have been performed with disparate success in animal models of diseases. Many cell types were partially successful in reverting some of the pathological anomalies observed in the grafted models. However, some of them presented important drawbacks, such as their poor tissue distribution, transient effect, maybe due to decreased GABA release over time [13–15], or in the case of ES cells, the lack of safety due to potential generation of teratocarcinomas [16, 17].

In the last decade, a better comprehension of how and where the cortical and hippocampal interneurons originate has led to use their neuronal precursors in transplantation [18, 19]. We currently know that most of the GABAergic interneurons in the cortex and hippocampus are mainly generated in two regions of the subcortical telencephalon, known as the caudal and medial ganglionic eminence (CGE and MGE), from where they migrate tangentially to their final destination in the cerebral cortex [19–22]. In the last years, several groups have reported regenerative works using these MGE-derived GABAergic precursors, with striking results [23–28]. At present, they represent the most promising cell-based therapeutic alternative for GABA-related diseases.

In this paper, we will summarize the main regenerative approaches using GABAergic grafts for the treatment of epilepsy and neurodegenerative disorders. These include the use of different sources of GABAergic precursors, with a special emphasis in the MGE-derived cells, and their transplant in several model organisms of disease. In addition, we will also describe the implications of the GABAergic grafts on the modulation of synaptic activity and circuitry plasticity of the host.

## 2. GABAergic Cell Therapy for Epilepsy

Epileptic seizures reflect a hyperexcitation in the brain, which is attributed to an imbalance between inhibitory and excitatory networks [6]. Given the close relationship between GABA and epilepsy [6, 8], antiepileptic drugs (AED) targeting the GABAergic system are traditionally the preferred treatment, presenting an acceptable efficacy [29, 30]. However, up to a third of patients continue to experience seizures on maximal tolerated drug therapy [31, 32]. Refractory epilepsy remains a large clinical problem, since surgical resection is only appropriate for a minority of patients [33, 34]. In the last decades, cell-based therapies using GABAergic grafts have emerged as an alternative treatment for epilepsy, since they may restore the lost equilibrium by cellular replacement of the missing/altered inhibitory neurons or modulating the hyperactive excitatory system [35–37]. The therapeutic strategies are multiple: general secretion of GABA, by the grafted cells to increase the seizure threshold, or specifically located in the focus of epilepsy and/or the areas responsible for seizure transmission to block it; direct replacement of malfunctioning or lost inhibitory interneurons; interaction of the transplanted GABAergic cells with activating system to modulate its plasticity, and levels of activity; finally, rewiring of aberrant excitatory fibers, such as mossy fiber in temporal lobe epilepsy (TLE), towards inhibitory GABAergic cells.

According to these strategies, several GABAergic cell types, with different origins and characteristics, have been assayed in animal models of epilepsy to evaluate their therapeutic potential. In the following sections, we describe in detail the most representative cell types and assays.

### 2.1. GABA-Producing Cells

A first therapeutic approach for epilepsy includes transplantation of fetal precursors from different brain regions, and cells genetically modified to produce and secrete GABA were transplanted into the hippocampus or in regions implicated in seizure generalization [9, 13–15, 38–41]. More than 20 years ago, Stevens et al. transplanted embryonic cerebellar and cortical tissue, rich in GABA or norepinephrine neurons, in the amygdala-kindled rat model of epilepsy [41]. Transplantation into the deep prepiriform area transiently raised seizure thresholds, showing for the first time that cell transplantation could be valuable for epilepsy treatment. Previously, Isacson et al. had already shown that transplantation of GABAergic cells from striatal primordia significantly ameliorates the lesion-induced locomotor hyperactivity in the ibotenic acid rat model of HD [10]. These seminal works strongly suggested that intracerebral grafting of inhibitory neurons may be an adequate strategy for seizure suppression [9]. Following this strategy, several groups isolated cells from the late striatal primordial (E14-15 in rats), to transplant them in regions thought to be critically involved in seizure propagation, such as the substantia nigra, as an effective means of permanently blocking seizure generalization in different models of epilepsy, mainly kindled rats [13, 14, 38–40]. All the kindling studies reported significant increase in afterdischarge thresholds and marked reduction in seizure severity compared with pretransplantation values [13, 17, 42]. In drug-induced models of epilepsy, these cells also suppressed the development of motor-limbic seizures and reduced the mortality rate [38–40]. However, the seizure-suppressing effect of GABAergic grafts was transient, likely due to progressive reductions in GABA levels. For this reason, it was attempted the development of immortalized glial and neuronal cell lines genetically engineered to produce GABA [37–39, 43, 44]. The ability to generate self-renewing clonal populations of transplantable GABA-producing cells provides an unlimited cell source and a good level of control on GABA production. GABA-producing cell lines demonstrated the ability to retard the development of seizures and block the expression of established seizures in kindling, kainic acid, and pilocarpine models of epilepsy [37–39, 43, 44]. However, these cell lines presented serious limitations that diminish their clinical potential [37]. The use of the SV40 large T oncogene for their immortalization raises concerns about tumorigenicity [38, 39]. In addition, in the host brain, a strong tissue reaction was reported including graft rejection, massive infiltration of inflammatory immune cells, and gliosis. Besides the concerns of tumorigenicity and immunogenicity, a major problem was the inability to sustain long-term effects due to the lack of survival or integration of the graft-derived cells [37]. To date, there is no report of engineered neuronal cells becoming fully differentiated and integrated into the seizure circuit of the host. This lack of integration may limit access to trophic factors and thus reduce the survival potential of these cells, and as a consequence, their mediated effects are transient.

### 2.2. Neuronal and Embryonic Stem Cells

The establishment of techniques that allow the isolation and culture of embryonic stem cells (ESCs), and neuronal stem cells (NSCs) from fetal and adult brain tissue, provided new sources of GABAergic cells for treating epilepsy to the scientific community [45, 46]. ESCs are isolated from the inner cell mass of the developing blastocyst and retain the ability to generate every cell type present in the body, including neurons [47, 48]. NSCs show a more restricted ability to generate only those cell types that constitute the nervous system; neurons, astrocytes, and oligodendrocytes [49]. NSCs can be expanded in culture using mitogens, mainly bFGF and EGF, which keep them in an undifferentiated state, forming floating cell aggregates, named neurospheres [50, 51]. Both cell types, ESCs and NSCs, are very promising in terms of providing an infinite supply of donor cells for neuropathological condition treatments. An additional advantage is the possibility to direct their differentiation toward specific cell types, in this case GABAergic neurons. In fact, default differentiation pathway for many SC lines in culture seems to be the GABAergic lineage [52, 53].

Despite these interesting properties, few studies report on the use of ESC and NSC in animal models of epilepsy [45, 46]. Rüschenschmidt et al. [54] reported that ESC-derived neuronal precursors (ESNPs) transplanted into the hippocampi of both control and pilocarpine-treated rats were able to generate action potentials and expressed voltage-gated Na^+^ and K^+^ currents, as well as hyperpolarization-activated currents. Anyway, electrophysiological activity and action potentials were lower than those in host neurons, typical of immature cells and suggesting an incomplete maturation process. Indeed, the grafted cells formed big clusters, and there was no evidence of cell type-specific differentiation one month after the transplant. In addition, no obvious difference was found between the functional properties of the transplanted cells in sham control and in pilocarpine-treated rats, and no improvement was described in the symptoms or electrophysiological activity of the epileptic rats after the transplants.

In contrast, Carpentino et al. [55] reported that grafted ESNP into the normal and kainic acid-treated mice partially migrated and differentiated towards neuroblasts and dentate granule neurons, or oligodendrocytes and astrocytes, depending on the brain area where they were finally located. However, some cells grafted in mice not subjected to seizures displayed a marked tendency to form tumors, and this effect was more pronounced in the dentate gyrus than in the fimbria. This suggested that seizures induce molecular changes that promote region-specific neural differentiation and suppress tumor formation. Finally, effects on the epileptic condition of the mice after ESNP transplantation were not reported, as well.

More recently, Shindo et al. [56] optimized a method to induce differentiation of GABAergic neurons from ESNP, and transplanted them into kindled epileptic mice to analyze a possible morphological and functional recovery. Two weeks after transplant, they observed a partial recovery of seizures. This was likely due to GABA production of transplanted cells, since histological analysis showed a high percentage of cells expressing GAD67. However, the morphology and cluster formation of the grafted cells suggest a lack of integration in the host circuitry.

From these reports, it is evident that the use of ESC in epilepsy treatment needs to be improved. Safer conditions to avoid tumorigenicity are necessary, and percentage and quality of differentiation toward GABAergic neurons should be increased. Improving the differentiation protocols from ESC and generating cell lines that are strongly committed to specific neural lineages in culture prior to grafting might be helpful. Several groups are working with this idea and have reported advances in the generation of GABAergic interneurons from ESC with high efficiency *in vitro* [57] and a good degree of functionality *in vivo* [58].

NSCs partially overcome some of the problems presented by ESC. They can be isolated from fetal or adult brain regions already committed in the generation of certain types of neurons; moreover, they prevent ethical issues and do not form tumors; and they could potentially be harvested in culture for prolonged periods, as neurospheres, to be used as a source of donor tissue for grafting [49–51]. Shetty et al. have isolated and cultured NSC from two fetal regions of the rat and grafted them in epileptic models. In a succession of reports using E19 hippocampal grafts or cultured NSC from this region [59–65], they reported the ability of this precursors to give rise to both hippocampal pyramidal-like neurons and interneurons in the host brain. However, barely more than 50% of transplanted neurospheres became differentiated cells, showing mainly an astrocyte phenotype and only in a small proportion a neuronal one. Integration of the transplanted cells was also poor; they form big clusters interfering with the normal hippocampal morphology. However, grafts located in or near the degenerated CA3 cell layer established commissural projections with the contralateral hippocampus. In addition, they revealed the capability of these grafts to restore disrupted hippocampal mossy fiber circuitry by attracting host mossy fibers sufficient to suppress the development of aberrant circuitry in hippocampus. The graft-induced long-term suppression of aberrant sprouting may provide a new avenue for amelioration of hyperexcitability [62].

Similarly to the previous reports, the Shetty's group transplanted striatal NSCs in the hippocampus of adult rats after status epilepticus induced by kainic acid [66]. The cells, pretreated with fibroblast growth factor-2 and caspase inhibitors, presented a good survival rate but limited ability to migrate, remaining close to the injection site. Nonetheless, a small percentage of these cells differentiated into GABAergic neurons and were able to reduce the seizure frequency in the kainic acid model of TLE.

Human NSC have been also tested in the pilocarpine-induced rat model of TLE [67]. They differentiated into cells that were positive for GABAergic (26%), glutamatergic (2%), or astrocytic (21%) markers. Grafted cells reduced the amplitude of extracellular field excitatory postsynaptic potentials in the hippocampal CA1, decreased the percentage of pilocarpine rats that developed spontaneous seizures, and declined both seizure frequency and severity.

### 2.3. MGE-Derived Neuronal Precursors

As mentioned in the introduction, in the last decade, the origin of cortical and hippocampal GABAergic interneurons has been elucidated [19–22]. Located in a restricted region of the ventral telencephalon known as the MGE and CGE, these precursors migrate long distances to cover the neocortex and hippocampal primordial where they complete their differentiation. In theory, these precursors should be good candidates for treating GABA-related diseases, since they are already committed to interneurons and migrate naturally long distance covering the brain parenchyma. They should overcome the difficulties presented by other sources of cells and achieve higher levels of inhibition or modulate the excitatory activity in the host. To verify this possibility, our group grafted fresh isolated, with no other manipulation, MGE-derived precursors into the neonatal normal brain [18]. MGE-derived cells gave rise to neurons that migrated, embracing wide areas of the cortical plate, striatum, and the hippocampus. More than 70% of the grafted cells differentiated into fully mature GABAergic interneurons, demonstrated by the expression of molecular markers such as calcium binding proteins. More importantly, electrophysiological analysis demonstrated these cells were able to integrate into the local circuits and make functional synapses with existing neurons, influencing the level of GABA-mediated synaptic inhibition. This was the first time that full mature electrophysiological activity and modulation of the host activity by GABAergic grafts was demonstrated. These observations strongly suggested the complete maturation of the grafted cells and its suitability for cell-based antiepileptic therapies.

In the following years, several groups tested these MGE-derived cells in different animal models of epilepsy. As proof of principle for a cell replacement therapy after lost or reductions in GABAergic neurons, our group grafted MGE-derived cells into a mouse model with a disinhibited brain environment caused by specific ablation of interneurons [28]. This was achieved by intrahippocampal microinjection of the neurotoxic Saporin conjugated with an analog of substance P (SSP-Sap), that selectively targets and eliminates the GABAergic interneurons expressing the substance P receptor, neurokinin-1 (NK-1) [68]. This experimental approach helped to address whether MGE-derived interneurons can integrate under neuropathological conditions and not only increase but also restore deficits in the inhibitory synaptic function as consequence of reductions in the number of GABAergic neurons. The specific GABAergic ablation leads to reductions in GABA-mediated synaptic inhibition, hyperexcitability, and increased susceptibility to pentylenetetrazol-induced seizures (PTZ), similarly to other models with reductions in interneurons [68–70]. MGE-derived cells in SSP-Sap-treated mice repopulate the hippocampal ablated zone with cells expressing molecular markers of mature interneurons. Similar to transplants in normal neonatal brain [18], the grafted MGE-derived cells migrated long distance covering the whole ablation area and acquired a fully mature morphology two months after transplantation with good survival rates (~25%). Immunohistochemical analysis revealed that more than 60% of graft-derived cells expressed GABA and specific molecular markers for mature interneuron subpopulations. Interestingly, electrophysiological analysis showed a restoration of the postsynaptic inhibitory current kinetics on CA1 pyramidal cells of ablated hippocampus after transplant, and more importantly, this was associated with reduction in seizure severity and decrease in postseizure mortality induced by PTZ [28] consistent with an enhancement of GABAergic inhibition after cell transplantation. In addition, these effects were stable over time. We performed a followup to 6 months after the transplant with similar results. This is logical, since we have verified MGE-derived cell survival more than 1 year after the transplant and, importantly, tumor formation was never detected.

MGE-derived cells are able not only to replace a loss of GABAergic neurons and reduce the mortality to PTZ-induced seizures, but also they show an intrinsic antiepileptogenic activity. Baraban et al. [23] grafted MGE-derived cells into neonatal Kv1.1 mutant mice, characterized by the lack of voltage-activated K^+^ channel, Kv1.1. These mice exhibit a high frequency of behavioural and electrographic seizures few weeks after birth. GABA-mediated synaptic and extrasynaptic inhibition onto host brain pyramidal neurons was significantly increased after bilateral transplant, and significant reductions in the total number, duration, and frequency of spontaneous electrographic seizures were observed. These findings suggest that MGE-derived interneurons could prevent and ameliorate abnormal excitability in infants. This is an interesting possibility, since MGE grafts may block generalization of seizures and improve life conditions in the patients. We have confirmed the anticonvulsant ability of these cells by maximum electroconvulsive shock (MES) assay after grafting in neonatal mice [24]. MES has remained one of the gold standards for AED screening [71]. The test evokes a single seizure applying a high-intensity current. Two months after transplantation in postnatal day 3 mice, MGE-grafted cells were able to protect against clonic seizures induced by MES, and a 5-fold reduction in the mortality rate was observed. This data strongly suggests that MGE grafts block the generalization of the seizures and allow a better control of the transition between tonic and clonic seizures. If we consider the MGE-grafts as a new AED, they perform better in MES assay than many AEDs already commercially available in the clinic.

However, before thinking of a clinical application of this cell type, some technical problems should be eliminated for instance, the limiting number of cells available for transplantation. One possibility is the amplification of MGE-derived cells in culture. MGE cells, cultured as neurospheres, have also been tested in the kainic acid model of TLE [27]. However, the interaction of MGE precursors with mitogens in culture seems to modify importantly their behaviour and neuronal commitment. MGE neurospheres gave rise mainly to astrocytes and only in a small proportion to GABAergic cells after transplantation. Nonetheless, these cells grafted into the hippocampi of adult rats restrained spontaneous recurrent motor seizures, with no improvement of the cognitive function. Authors suggested that expression of GDNF by more than 50% of the grafted cells may underlie the therapeutic effect of MGE-NSC grafts, given the role in seizure suppression of this neurotrophic factor [72]. 

Taken together all these works, we have a scenario where MGE-derived cells are the most promising source of GABAergic neurons for cell-based therapies. However, before clinical application, we should continue studying the possible implication of the interaction of new grafted cells with those from the host; their modulation of synaptic activity, maybe by modifying neuronal plasticity; the possible consequences on behaviour. We will review these implications in the following section. However, we should have in mind that epilepsy etiology is multiple, and in consequence not all of the epilepsies should response equally to MGE-derived interneuron grafts. In addition, we should be cautious. Certain types of GABAergic interneurons together with aberrantly behaving excitatory pyramidal neurons in the subicular region of the hippocampus can precipitate epileptic seizures instead to stop them [73]. In keeping with this idea, it has been also reported a role of GABA-mediated signaling in ictogenesis, contributing to epileptiform synchronization that lead to the generation of electrographic ictal events in the cingulated cortex and limbic areas of the brain [74, 75]. Therefore, grafting of certain subclass of GABA-producing cells in a wrong location in some epilepsy types may lead to seizure exacerbation.

Before clinical application, we should continue exploring the effects of the grafts on several animal models of epilepsy with different etiologies; study the possible implication of the interaction of new grafted cells with those from the host; their modulation of synaptic activity, maybe by modifying neuronal plasticity and the possible consequences on behaviour. We will review these implications in the following sections.

## 3. GABAergic Grafts for Parkinson's Disease (PD) and Stroke

PD is triggered by the loss of mesencephalic dopaminergic neurons localized in the substantia nigra pars compacta (SNpc). This cellular loss eliminates dopaminergic projections to the striatum and their supply of dopamine which modulates striatal-dependent extrapyramidal motor behaviour. Therefore, PD patients experience motor dysfunctions including tremor, rigidity, bradykinesia, and postural instability. Alleviation of motor symptoms is obtained by the administration of the dopamine precursor L-DOPA; however, its prolonged use over years induces the development of severe side effects known as dyskinesia (abnormal involuntary movements) that only in part are mitigated by different regimens of pharmacological coadiuvants. 

In addition, many attempts of gene and cell-based therapies are in progress to establish treatments that can be complementary and additive to the standard pharmacological approach. In particular, a gene therapy approach has been developed to deliver the glutamic acid decarboxylase (*GAD*) gene, catalysing the synthesis of GABA, directly into neurons of the subthalamic nucleus [76]. In PD, activity of the subthalamic nucleus (STN) is increased mainly because of reduced GABAergic input from the globus pallidus. Interestingly, the focal delivery of GAD in the STN contributed to reducing its overactivity leading to an amelioration of the clinical neurological symptoms. Safety and tolerability of this gene therapy protocol has been demonstrated in a phase I trial although with a reduced number of patients and over a limited period of time (1 year) [77]. Thus, promoting GABAergic neuronal activity in specific nuclei can contribute in restoring a balance in the basal ganglia neuronal outflow controlling the extrapyramidal motor system. 

In addition to these approaches, the clinical impact of cell replacement has been evaluated in animals and humans over the last two decades. These approaches are aimed to reconstitute a local dopaminergic network capable of a feedback controlled dopamine release upon delivering of dopaminergic neurons in the affected striatal compartment. A similar procedure has been pioneered in humans using cellular grafts of fetal nigral tissues [78–80]. This approach led to some important symptomatic improvements, however, often associated with the development of extremely severe dyskinesia [81]. These side effects are probably due to the high heterogeneity of the grafted tissue containing only a minimal fraction of dopaminergic precursors (5%) in an overall population of different cell types including serotonergic and GABAergic neurons [82, 83]. Recently, an alternative strategy of cell transplantation has been validated in a PD animal model. This is based on transplanting GABAergic precursors isolated from the embryonic MGE/CGE into the adult striatum [84]. At first glance, this approach introducing inhibitory neurons in a tissue already deprived of dopamine might seem counterintuitive. However, thinking of PD as an activity outflow unbalance among different striatal neuronal networks, this methodology finds a strong rationale [85]. Noteworthy, E14.5 MGE/CGE cells injected into a single site were able to migrate throughout the striatal tissue, but not beyond it. The extent of migration is similar to that described for these cells upon transplantation into the adult cortex (see above). Therefore, MGE/CGE GABAergic neuronal precursors have a tendency to actively disperse within different adult brain tissues, and this represents a strong attractive feature for an efficient cell-based therapy. However, this should not be considered of general rule, since the same cells grafted in the subthalamic nucleus are unable to migrate from the injection site [84]. Possibly, this is the case since cortex and striatum, contrary to thalamic areas, are the forebrain regions which are normally colonized by these cells during embryogenesis and might retain some molecular or structural cues that allow this behaviour even in adulthood. Are these cells able subsequently to survive, mature, and integrate in the host striatal tissue? Martínez-Cerdeño and colleagues found that despite the great majority of the MGE/CGE-transplanted cells which were lost after 1 year from transplantation, 1% of them survived and presented morphological and functional features of mature interneurons of the three major subtypes, namely, calretinin+, parvalbumin+, and somatostatin+ cells [84]. Therefore, only a minimal fraction of MGE-transplanted cells are able to survive for long time in the striatal tissues and this probably reflects the need for establishing stable and functional connections with the host neuronal network for promoting their survival. Remarkably, even though the transplanted interneurons accounted for only about 5% of the total endogenous GABAergic neuronal population, they were sufficient to elicit a significant motor and behavioural recovery in the 6-hydroxydopamine-lesioned rats. How might this occur? The authors revealed the integration of the grafted interneurons by showing the formation of de novo synapses with the host neurons, and hence, they suggest that it is the graft-mediated reorganization of the basal ganglia network that fosters the functional recovery observed [84]. In fact, the striatum is the key centre of the extrapyramidal tract which controls thalamic efferents to the motor cortical regions. This circuitry is organized in two main neuronal assemblies known as the direct and the indirect pathways. The first connects striatum-internal globus pallidus and thalamus and activates thalamic activity. The second restrains thalamic activity and is connecting striatum-internal and -external globus pallidus and thalamus. Hence, these two pathways converge to the thalamus as their final target centre and regulate its activity by playing reciprocal opposing functions. In PD, dopamine depletion in the striatum produces two concomitant effects. First, it reduces the activity of the direct pathway while promoting the indirect pathway creating an upraised inhibitory outflow to the thalamus. 

One plausible mechanism by which MGE-transplanted cells promote a symptomatic relief in PD animals is to restore a balance in the total output of these two pathways over the thalamus by preferentially inhibiting the indirect pathway [85]. Although this explanation needs more experimental evidences, nonetheless these studies reveal how a small transplanted population of interneurons has the capability to modulate the plasticity of long-ranging and complex neuronal circuitry and restore a functional unbalance between related neuronal systems. 

Recently, similar cell transplantations of embryonic MGE GABAergic precursor cells have been carried out in a mouse model of stroke [86]. Focal ischemia in cortical and nearby striatal areas was produced by middle cerebral artery occlusion, and embryonic MGE cells were transplanted in multiple sites in adjoining regions. Noteworthy, MGE-transplanted animals improved in their locomotion and motor coordination with a significant improvement in both tests respect to sham-injected controls [86]. Similar to previous studies, embryonic MGE cells developed in fully mature neurons featuring spontaneous action potentials and connecting to host neurons. However, the amount of MGE grafted cells that differentiated into mature neurons after 4 weeks from transplantation were only a limited fraction accounting for 20% of the total. Surprisingly, the rest of the cells resulted negatively for astrocyte or oligodendrocyte markers indicating that the transplanted cells remain blocked to a progenitor state unable to complete the differentiation in any cell lineage [86]. This is in striking contrast with the differentiation behavior of grafted MGE cells in the other disease murine models previously described [82]. 

Nonetheless, the authors noted that the MGE cell grafting stimulates axonal reorganization of the host tissue [86]. In fact, the axonal sprouting and neurite reorganization in the injured site was strongly increased after cell transplantation. These results suggest that grafted MGE neurons might stimulate endogenous repairing mechanisms or formation of alternative neuronal assemblies to support the functions of the lost tissue. To which extent exogenous MGE cells can trigger neuronal rewiring and plasticity of the host tissue remains to be better exemplified. To be noted, also MGE cell graftings in PD animal models induced some changes in the host tissue as for instance the re-expression of the calcium-binding proteins calretinin and calbindin by host striatal cells nearby the transplantation site [84]. The changes might also be promoted by all sorts of trophic factors released by the grafted GABA neuronal precursors that can stimulate neuritogenesis or synaptic connections.

Although many questions remain unanswered, transplantation of embryonic MGE GABAergic cells has resulted surprisingly effectively in promoting clinical improvements in animal models for different chronic or acute neurological disorders. These results call for a better understanding of the cellular and molecular mechanisms by which the MGE grafts can promote this positive outcome. One of them may be the increased delivery of GABA neurotransmitter. To date, direct biochemical measure of GABA concentrations after MGE-derived grafting has not been reported. However, patch clamp analysis of spontaneous postsynaptic inhibitory currents in projection neurons of the grafted area strongly suggests a direct effect on their frequency and amplitude, mediated specifically by presynaptic GABA delivery from the transplanted cells [18, 28]. One other mechanism may be secondary to release of trophic factors by the grafted cells. GDNF has been reported to be secreted by the astrocytes cotransplanted with the MGE cells [27], what improve epileptic condition. We cannot discard the delivery of some other neurotrophic factors that stimulate endogenous repairing mechanisms, or even neurogenesis, as mentioned above. Further efforts should be devoted to decipher the multiple mechanisms implicated in the functional outcomes mediated by GABAergic cell transplants, including modulation of endogenous plasticity.

## 4. MGE GABAergic Cellular Grafts Induce Cortical Plasticity

Cortical circuits are sensitive to experience during well-defined intervals of early postnatal development called critical periods [87, 88]. After the critical period, plasticity is reduced or absent. Monocular deprivation (MD) is a classic model of experience-dependent plasticity. In the mammalian binocular visual cortex, neurons are activated to different degrees by visual stimuli presented to one eye or the other, a property called ocular dominance (OD). If vision is normal for both eyes during development, the majority of visual cortical neurons are binocular. If one eye is occluded during development, visual cortical neurons become dominated by the nondeprived eye. This change in OD is taken as a sensitive index of plasticity of visual connections. OD plasticity is particularly high during a critical period of postnatal development and declines with age [89, 90].

 Accumulating evidence supports a pivotal role for late-developing excitation versus inhibition circuit balance in the initiation of sensitive periods. For example, the onset of visual cortical plasticity is delayed by genetic disruption of GABA synthesis [91, 92]. Conversely, the application of benzodiazepines or other treatments that accelerate GABA circuit function triggers premature plasticity [93]. Therefore, the onset of OD plasticity is triggered by the establishment of a functional network of inhibitory synaptic transmission. Southwell and colleagues asked whether transplantation of MGE GABAergic precursors could be sufficient to trigger a plasticity respond in the host cortex [94]. In mice, OD plasticity reaches a peak in the fourth postnatal week, when cortical inhibitory neurons are 33–35 days old. Thus, the authors transplanted E13.5 mouse MGE GABAergic precursors in perinatal or early-postnatal brains and ascertained the induction of visual plasticity in the host brains [94]. Grafted MGE cells differentiated into GABAergic interneurons with a great efficiency and only 0.2% of them developed morphology of astrocytes. Remarkably, the cellular graftings were able to successfully trigger visual plasticity in the host [94]. However, this induction was achieved only for a short period of time which correlated with the age of the transplanted cells. In fact, transplantation was effective when the cells were 33–35 days while soon later at 43–46 days old the same cells failed to trigger the same effect. Therefore, the cellular age of the transplanted population strictly determines the effects on cortical plasticity.

The introduction of a supplemental amount of inhibitory interneurons would suggest that an increased inhibitory tone is the trigger for such neural rejuvenation. However, this is not the case since pharmacological enhancement of inhibition does not induce similar effects [95]. The answer is rooted probably in the nature of the synaptic contacts established by grafted interneurons with the host neuronal circuitry. Indeed, transplanted inhibitory neurons form weak but numerous synaptic connections with neighbouring excitatory neurons in the host brain. Thus, these new connections are believed not to simply raise the general inhibitory tone, but rather to promote an overall reorganization of the cortical circuitry by introducing a new set of weak inhibitory synapses. This pattern of newly established synaptic contacts represent an ideal biological substrate capable of enhancing the Hebbian plasticity mechanisms during the critical period [96]. 

It is noteworthy that the grafted interneurons promote plasticity only when they reach a cellular age comparable to that of the endogenous counterpart during the critical period. This data strongly suggest that plasticity is successfully initiated by a cell-autonomous program endowed in interneuron progenitors which is minimally influenced by the age of the host tissue. These findings open a new scenario where cell transplantation might be effective in reprogramming neural activity up to triggering plasticity processes. Nonetheless, a number of questions need to be answered to understand the safety and efficacy of this procedure. For instance, (i) whether transplantations of interneurons are able to induce visual plasticity even in the adulthood, (ii) if the grafted animals display any neuronal misbehaviour at later stages triggered by the action of the transplanted cells, (iii) if plasticity is promoted by a specific class of interneurons, and (iv) the assessment of the minimal number of cells to be grafted for inducing brain plasticity.

Although the transplanted-induced plasticity lasts for few days, this might be sufficient to trigger long-lasting neural circuitry reorganization. On this view, this procedure opens the exciting opportunity to induce or facilitate the restoration of normal function in injured or degenerative disorders. Future studies are warranted to assess the regenerative potential of this approach in the developing and adult-diseased brains.

## 5. New Sources for MGE GABAergic Neurons

Considering the findings described above, MGE GABAergic neurons exhibit properties well suited for therapeutic applications in seizures and other neuropsychiatric and neurodegenerative diseases. However, to explore such possibility, it is necessary to identify a renewable source for these cells compatible with their preclinical exploitation. An interesting possibility is generating these cells from *in vitro* differentiation of embryonic or somatic neural stem cells (ESCs and NSCs, resp.). NSCs can be isolated from mouse and human neural tissues and can be propagated for long time in cultures as neurospheres or in adhesive conditions [97–100]. Upon differentiation, NSCs generate a mixed population of GABAergic and glutamatergic neurons, whose ratio is dependent on specific growth culture and differentiation conditions [97, 101]. However, these cells show generally poor developmental plasticity. In fact, after prolonged time in culture, they retain only in part the molecular regional code identity of the area from which they originate and result generally resistant to be coaxed to other neuronal subtypes [102–104]. In contrast, numerous results have shown how ESCs can be converted efficiently in various neuronal subtypes. In particular, some procedures have been recently proposed for directing ESC differentiation into cortical GABAergic interneurons. In an elegant set of experiments, Danjo and colleagues refined the timing and concentrations of Sonic Hedgehog (Shh) stimulation for inducing ESCs neural ventralization and generating either LGE or MGE progenitors [105]. In the latter case, ESC-derived MGE progenitors displayed the ability to migrate and distribute into the developing cortex generating GABAergic interneurons. Interestingly, the authors further showed how a diverse source of FGF signalling can alternatively select for an MGE- or CGE-derived GABAergic cell fate. These results set the experimental conditions to generate different subtypes of cortical GABAergic interneurons with specific electrophysiological and connectivity properties. Further, a different study showed the ability of ESC-derived MGE progenitors to complete their maturation once transplanted *in vivo* generating functional cells with physiological and neurochemical characteristic of GABAergic cortical interneurons [106]. These findings lay the ground for testing the potential of ESC-derived GABAergic interneurons to treat preclinical model of neurological disorders upon direct cell transplantation.
